# A retrospective population-based analysis of 19,640 pediatric and young adult males with gynecomastia

**DOI:** 10.1016/j.jpra.2026.03.025

**Published:** 2026-03-25

**Authors:** Daniel Hilewitz, Gal Ben Tsion, Dean Ad-El, Sagit Meshulam-Derazon, Eyal Kalish, Asaf Olshinka

**Affiliations:** a Gray Faculty of Medicine, Tel Aviv University, Tel Aviv, Israel; b Division of Plastic Surgery, Rabin Medical Center, Petah Tikva, Israel; c Pediatric Plastic Surgery Unit, Schneider Children's Medical Center, Petah Tikva, Israel

**Keywords:** Pediatric gynecomastia, Idiopathic, Obesity, Drug-induced, Pathology-associated, Surgical intervention

## Abstract

Gynecomastia is the most common breast condition in pediatric and young adult males, yet large-scale studies evaluating its etiologies, risk factors, and management remain scarce. In adolescents, gynecomastia is most frequently idiopathic or obesity-related, while secondary causes are rarer. Persistent presentations may cause psychosocial distress and lead to surgical correction, though the optimal diagnostic approach remains controversial. We conducted a retrospective, population-based study of 19,640 males aged 10–25 years with documented gynecomastia diagnosis, extracting demographic and clinical variables including age, body mass index (BMI) Z-scores, family history, comorbidities, medication exposure, and surgical status. Reporting was in accordance with the STROBE guidelines. Most patients were idiopathic (74.3%), followed by medication-related (23.1%) and pathology-associated (3.4%). For patients with pathology-associated cases compared with other etiologies, the mean age and mean BMI Z-score were higher, and surgeries were more frequent (all *p* < 0.001). The prevalence of pathology increased with age, whereas obesity-related and familial cases predominated in younger children. Morbid obesity was significantly associated with higher surgical rates (5.2% vs. 2.1% in non-obese, *p* < 0.001). Independent associations with surgical intervention included older age (odds ratio (OR 1.20), higher BMI Z-score (OR 1.30), ≥2 affected siblings (OR 8.35), and testicular tumors (OR 14.06). Model discrimination (area under the curve = 0.973) was excellent. In conclusion, gynecomastia in younger boys is primarily idiopathic or obesity-related, while older adolescents are more often diagnosed with secondary causes. Age- and risk-tailored diagnostic strategies, including early screening for obesity, family history, and medication exposure may optimize management and guide surgical decision-making.

## Introduction

Gynecomastia, defined as the benign proliferation of glandular breast tissue in males, is the most common breast condition in this population.^1^ The condition is broadly classified as physiological or non-physiological. Physiological gynecomastia typically occurs at pubertal and older adult stages, affecting up to 50–60% of adolescents, and 65% of older men.^2^ A positive family history may suggest genetic predisposition, with up to 50% of adolescent males reporting a father or sibling with gynecomastia.^3^

Obesity substantially influences the clinical presentation of gynecomastia. Up to 50% of affected adolescents are with obesity, many with pseudo-gynecomastia—breast enlargement due to adipose rather than glandular tissue.^4^ A pediatric review found about half of adolescents with pubertal gynecomastia had elevated body mass index (BMI) Z-scores (>1). This underscores a strong association between obesity and glandular proliferation.^5^ Another study demonstrated higher gynecomastia prevalence among high-school males with obesity, which was attributed to increased aromatase activity and leptin-mediated breast stimulation.^4^

Non-physiological gynecomastia arises from diverse secondary causes, including hypogonadism, hyperthyroidism, inflammatory bowel disease (IBD), and medications such as cardiovascular, psychotropic, or endocrine agents, as well as dietary supplements or illicit substances.^2^^,^^6^^,^^7^ Rarely, the condition indicates a hormone-secreting neoplasm, necessitating thorough evaluation.^5^ While physiological gynecomastia is generally self-limiting, persistent or symptomatic presentation can cause psychosocial distress, cosmetic concerns, and physical discomfort, often prompting surgical correction.^8^ However, optimal diagnostic criteria and the need for preoperative hormonal or imaging assessments in otherwise healthy adolescents remain debated.^9^ Distinguishing idiopathic from reversible secondary etiologies is a critical clinical challenge.

Despite the high prevalence of gynecomastia, large-scale population-based studies in children and young adults remain scarce. This study aimed to characterize demographic and clinical factors associated with idiopathic versus secondary gynecomastia. We focused particularly on modifiable contributors such as BMI and medication exposure, to identify associations with surgical intervention.

## Methods

### Eligibility criteria and data collection

This retrospective study was performed at Schneider Children’s Medical Center, a tertiary pediatric hospital within Clalit Health Services, Israel’s largest healthcare provider. Following Helsinki ethics approval (0507-23-RMC), de-identified data were retrieved from Clalit Health Service’s hospital and community clinic records. Only the final analytic dataset was provided. All the records were anonymized and assigned serial numbers to ensure confidentiality. Gynecomastia was diagnosed based on a physical examination by a pediatrician, family doctor, plastic surgeon, general surgeon, or pediatric endocrinologist; and classified according to the Simon grading system. The study design, setting, clinical environment, and timeframe (January 2000–December 2023) were predefined to ensure comprehensive capture of all the pediatric and young-adult gynecomastia presentations across the Clalit Health Services network.

Inclusion criteria were males aged 10–25 years with a recorded diagnosis of gynecomastia during 2000–2023. The age range of 10–25 years was intentionally selected to capture pediatric and young-adult presentations of gynecomastia. Consequently, etiologies predominantly seen in older men fall outside the scope of this study. Exclusion criteria included: female patients, patients with incomplete age data, and duplicate encounters. When duplicates existed, only the latest documented diagnosis was included. The demographic and clinical data collected included the age at diagnosis and body mass index (BMI) Z-scores. The latter were calculated using age- and sex-specific standardized growth curves. The categories were defined as normal weight (0 < *Z*-score <1), obesity (1 < *Z*-score <2), and morbid obesity (Z-score ≥2). Family history of gynecomastia (father or sibling affected) was documented.

Relevant comorbidities and medication exposure were recorded; medications were considered contributory if initiated at least one month before a gynecomastia diagnosis. Underlying systemic and endocrine disorders (e.g., hypogonadism, hyperthyroidism, IBD, and hormone-secreting tumors) were classified as secondary etiologies. For patients with overlapping pathology and medication exposure, etiologies were classified according to the underlying pathology. Comorbidities, pathology diagnoses, and medication classes were extracted using International Classification of Diseases 10 diagnostic codes and Anatomical Therapeutic Chemical medication codes from Clalit Health Service’s electronic medical records system. Surgical interventions included open glandular excision, closed excision techniques, liposuction, and combined procedures; of which the vast majority were performed by board-certified plastic surgeons. For all the patients, the indication for surgery was not medical necessity, but rather, age above 16 years, patient request (and parental approval for those under age 18 years), and the absence of contraindications. Postoperative outcomes beyond the actual performance of surgery were not available in the dataset.

### Statistical analysis

Continuous variables were summarized as mean ± standard deviation, and categorical variables as frequencies and percentages. Normality of distributions was assessed using the Kolmogorov–Smirnov test, and homogeneity of variances with Levene’s test. Group comparisons employed the Student’s *t*-test for normally distributed data and the Mann–Whitney U test for non-normally distributed data. Associations between categorical variables were analyzed using the Chi-square or Fisher’s exact test, as appropriate. Multivariable logistic regression was applied to examine associations of potential risk factors with (1) surgical intervention and (2) gynecomastia etiology (idiopathic vs. secondary). Variables that yielded *p* < 0.05 in univariate analyses or that were deemed clinically relevant were included in the models. Models were evaluated using receiver operating characteristic curves and corresponding areas under the curve. Missing data were <1% for all the variables and were handled using complete-case analysis. Sensitivity analyses were not required due to minimal missingness. Given the large, population-based dataset, advanced big-data statistical methods were required to ensure robust analysis. All the analyses were performed using SPSS Statistics (IBM Corp., Armonk, NY, USA). A two-tailed *p* ≤ 0.05 was considered statistically significant.

The data were reported and the manuscript was prepared in accordance with the STROBE (Strengthening the Reporting of Observational Studies in Epidemiology) guidelines (See the supplementary file).

## Results

### Study population

**Figure 1:**
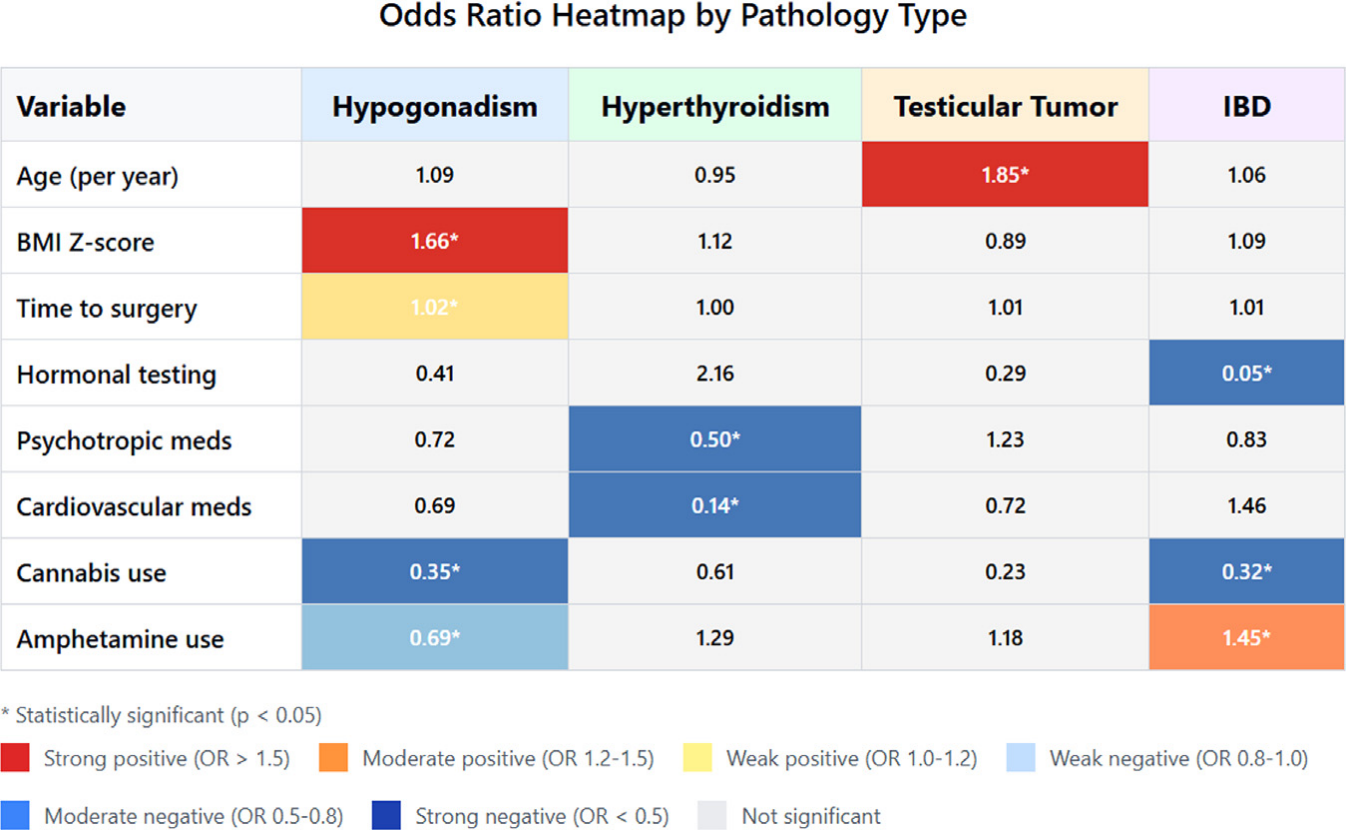
Heat map of the associations of the specific pathologies with the characteristics assessed.

The study included 19,640 males diagnosed with gynecomastia. The mean age at diagnosis was 15.60 ± 3.56 years, the mean BMI was 24.29 ± 2.69 kg/m², and the mean BMI Z-score 0.91 ± 1.45. Most patients were idiopathic (*n* = 14,603; 74.3%). Medication-associated gynecomastia occurred in 4541 (23.1%). Pathology-associated gynecomastia such as hyperthyroidism, hypogonadism, or IBD; or pituitary, adrenal, or testicular tumors was found in 667 patients (3.4%). Among the pathology group, 160 (23.9%) had concurrent medication exposure. Table 1 details population characteristics by age group. Figure 1 presents a heat map of the associations of the specific pathologies with the characteristics assessed.

**Table 1:** Demographic and clinical characteristics stratified by age group.

Age groups, years		10–11 (*N* = 2022)	12–13 (*N* = 6221)	14–16 (*N* = 5987)	17–19 (*N* = 2530)	20–25 (*N* = 2880)
Age at diagnosis (years) Mean ± SD		11.30 ± 0.54	13.05 ± 0.55	15.28 ± 0.87	18.08 ± 0.80	22.61 ± 1.31
BMI, Mean ± SD		23.29 ± 2.45	23.60 ± 3.16	24.0 ± 1.30	25.94 ± 1.85	25.69 ± 0.49
BMI z-score. Mean ± SD		1.46 ± 1.47	0.87 ± 1.47	0.82 ± 1.49	0.82 ± 1.43	0.89 ± 1.26
Obesity (BMI Z-score >1)		1299 (64.2)	3020 (48.5)	2841 (47.5)	1163 (46)	1297 (45)
Family history of gynecomastia						
	Father with gynecomastia	26 (1.3)	104 (1.7)	99 (1.7)	47 (1.9)	67 (2.3)
	1 sibling with gynecomastia	59 (2.9)	227 (3.6)	306 (5.1)	108 (4.3)	108 (3.8)
	>2 siblings with gynecomastia	8 (0.4)	14 (0.2)	24 (0.4)	10 (0.4)	8 (0.3)
Alcohol use		3 (0.1)	9 (0.1)	9 (0.2)	11 (0.4)	21 (0.7)
Drug use		0 (0)	3 ()	4 (0.1)	4 (0.2)	20 (0.7)
Comorbidities		53 (2.6)	154 (2.5)	183 (3)	129 (5.1)	166 (5.8)
	Hypogonadism	29 (1.4)	51 (0.8)	89 (1.5)	61 (2.4)	70 (2.4)
	IBD	15 (0.7)	71 (1.1)	62 (1)	36 (1.4)	53 (1.8)
	Hyperthyroidism	8 (0.4)	26 (0.4)	25 (0.4)	21 (0.8)	33 (1.1)
	Testicular tumor	0 (0)	5 (0.1)	7 (0.1)	10 (0.4)	8 (0.3)
	Pituitary tumor	0 (0)	0 (0)	0 (0)	1 ()	2 (0.1)
	Adrenal tumor	1 ()	1 ()	0 (0)	0 (0)	0 (0)
Medications						
	Hormonal and endocrine	7 (0.3)	16 (0.3)	22 (0.4)	41 (1.6)	87 (3)
	Psychotropic	50 (2.5)	187 (3)	255 (4.3)	141 (5.6)	199 (6.9)
	Cardiovascular	1 ()	11 (0.2)	14 (0.2)	11 (0.4)	17 (0.6)
	Chemotherapeutic	1 ()	0 (0)	0 (0)	1 ()	3 (0.1)
	Amphetamine and methamphetamine	360 (17.8)	1228 (19.7)	1369 (22.9)	527 (20.8)	429 (14.9)
	Cannabis	8 (0.4)	24 (0.4)	34 (0.6)	17 (0.7)	29 (1)
Hormonal tests		1829 (90.5)	5585 (89.8)	5555 (92.8)	2407 (95.1)	2736 (95)
Months from diagnosis to surgery		0.96 ± 7.39	0.67 ± 5.88	0.85 ± 6.05	0.98 ± 6.74	0.80 ± 5.37

IBD, inflammatory bowel disease.

A positive family history was reported in 1215 patients (6.2%): 905 (74%) were idiopathic, 263 (21%) medication-related, and 47 (4%) pathology-associated. Although not statistically significant, familial clustering predominated in patients who were idiopathic. Table 2 presents the distribution of idiopathic gynecomastia by family history and age group, and shows the chances of a pathology-associated etiology.

**Table 2:** Distribution of idiopathic gynecomastia by family history and age group-odds for other pathology.

Age group, years			10–11	12–13	14–16	17–19	20–25
N			2022	6221	5987	2530	2880
Idiopathic gynecomastia (no drugs, medications, obesity) (N)			594 (29.4)	2522 (40.5)	2373 (39.6)	1052 (41.6)	1239 (43.0)
	Family history		15 (2.5)	115 (4.5)	156 (6.6)	64 (6.1)	73 (5.9)
		Overall pathology	0	2 (1.7)	3 (1.9)	3 (4.5)	2 (2.7)
		IBD	0	2 (1.7)	2 (1.3)	1 (1.5)	1 (1.3)
		Hyperthyroidism	0	0	0	1 (1.5)	1 (1.3)
		Hypogonadism	0	0	1 (0.6)	1 (1.5)	0
		Testicular tumor	0	0	0	0	0
	No family history		579 (97.5)	2407 (95.5)	2217 (93.4)	988 (93.9)	1166 (94.1)
		Overall pathology	7 (1.2)	34 (1.4)	48 (2.2)	46 (4.7)	55 (4.7)
		IBD	4 (0.7)	24 (1)	25 (1.1)	20 (2)	24 (2)
		Hyperthyroidism	2 (0.3)	7 (0.3)	5 (0.2)	6 (0.6)	14 (1.2)
		Hypogonadism	1 (0.2)	3 (0.1)	13 (0.6)	15 (1.5)	15 (1.3)
		Testicular tumor	0	0	5 (0.2)	5 (0.5)	2 (0.2)

The data are presented as absolute numbers, with percentages in parentheses.

IBD, inflammatory bowel disease.

### Pathology versus non-pathology gynecomastia

Table 3 compares patients with pathology-associated gynecomastia to those without underlying pathologies. Compared to patients without pathologies, for those with pathology-associated etiologies, the mean age was older (16.76 ± 3.95 vs. 15.56 ± 3.54 years; *p* < 0.001) and the mean BMI Z-score was higher (1.29 ± 1.59 vs. 0.89 ± 1.44; *p* < 0.001). Surgery was more frequent among patients with pathology-associated gynecomastia (6.4% vs. 3.3%; *p* < 0.001). Pathology likelihood increased with age: 2.5–3% for ages 10–16 years versus 5.1–5.8% for ages 19–25 years. Age was found to be associated across conditions. Compared with ages 20–25 years, for younger groups, IBD-related gynecomastia was markedly less frequent (10–11 years: odds ratio [OR] 0.391, *p* < 0.01; and 12–13 years: OR 0.623; 14–16 years: OR 0.582). Similar trends were seen for hypogonadism (12–13 years: OR 0.316, *p* < 0.001; 14–16 years: OR 0.606, *p* = 0.002) and hyperthyroidism (12–13 years: OR 0.378, *p* < 0.001; 14–16 years: OR 0.366, *p* < 0.001). Testicular tumor–related gynecomastia was more frequent in younger patients (12–13 years: OR 0.258, *p* = 0.021).

**Table 3:** Demographic, clinical, and etiological characteristics of patients with gynecomastia, stratified by those with and without background pathology.

Characteristics		Pathology (*N* = 667)	Non-pathology (*N* = 18,973)	*p* value
Age at diagnosis (years), Mean ± SD		16.76 ± 3.95	15.56 ± 3.54	<0.001
BMI, Mean ± SD		26.64 ± 1.44	24.21 ± 2.19	0.005
BMI Z-score. Mean ± SD		1.29 ± 1.59	0.89 ± 1.44	<0.001
Family history of gynecomastia				
	Father with gynecomastia	13 (1.9)	330 (1.7)	0.684
	1 sibling with gynecomastia	31 (4.6)	777 (4.1)	0.480
	>2 siblings with gynecomastia	3 (0.4)	61 (0.3)	0.480
Alcohol use		0 (0)	53 (0.3)	0.265
Drug use		2 (0.3)	29 (0.2)	0.284
Hormonal tests		666 (99.8)	17,446 (92)	<0.001
Surgery		43 (6.4)	633 (3.3)	<0.001
Surgery after diagnosis, months		2.07 ± 10.17	0.77 ± 5.96	<0.001
Medications		185 (27.7)	4313 (22.7)	0.003
	Hormonal and endocrine	14 (2.1)	159 (0.8)	<0.001
	Psychotropic	44 (6.6)	788 (4.2)	0.002
	Cardiovascular	7 (1)	47 (0.2)	<0.001
	Chemotherapeutic	0 (0)	5 ()	0.999
	Amphetamine and methamphetamine	146 (21.9)	3767 (19.9)	0.196
	Cannabis	11 (1.6)	101 (0.5)	<0.001

### Sub-analysis: etiologic groups

For patients with pathology compared to medication and idiopathic etiologies, the mean age was older (16.78 ± 3.95, 15.68 ± 3.44, and 15.52 ± 3.56 years, respectively; *p* < 0.001), and the mean BMI and BMI Z-scores were higher (Table 4). For the pathology group, surgery was more common (6.5% vs. 3.4% and 3.3%; *p* < 0.001) and the mean diagnosis-to-surgery interval was longer (2.1 ± 10.24 months vs. 0.77 ± 5.77 and 0.77 ± 6.01; *p* < 0.001). Drug use was more frequent among those with pathology and medication etiologies than among those with idiopathic etiologies (0.3%, 0.5%, and 0.1%, respectively, *p* < 0.001). Self-reported Alcohol use was slightly higher in the medication-related group (*p* = 0.002).

**Table 4:** Demographic, clinical, and etiologic characteristics of patients with gynecomastia, stratified by background pathology, medication use and idiopathic etiologies.

Characteristics		Pathology (*N* = 667)	Medications (*N* = 4381)	Idiopathic (*N* = 14,603)	*p* value
Age at diagnosis (years), Mean ± SD		16.78 ± 3.95	15.68 ± 3.44	15.52 ± 3.56	<0.001
BMI, Mean ± SD		26.64 ± 1.43	24.21 ± 1.38	24.21 ± 2.38	0.019
BMI Z-score. Mean ± SD		1.29 ± 1.59	0.93 ± 1.5	0.89 ± 1.43	<0.001
Family history of gynecomastia					
	Father with gynecomastia	13 (2)	76 (1.7)	254 (1.7)	0.896
	1 sibling with gynecomastia	31 (4.7)	172 (3.9)	605 (4.1)	0.593
	>2 siblings with gynecomastia	3 (0.5)	15 (0.3)	46 (0.3)	0.803
Alcohol use		0 (0)	22 (0.5)	31 (0.2)	0.002
Drug use		2 (0.3)	20 (0.5)	9 (0.1)	<0.001
Surgery		43 (6.5)	151 (3.4)	482 (3.3)	<0.001
Surgery after diagnosis, months		2.1 ± 10.24	0.77 ± 5.77	0.77 ± 6.01	<0.001

### Surgical intervention

**Figure 2:**
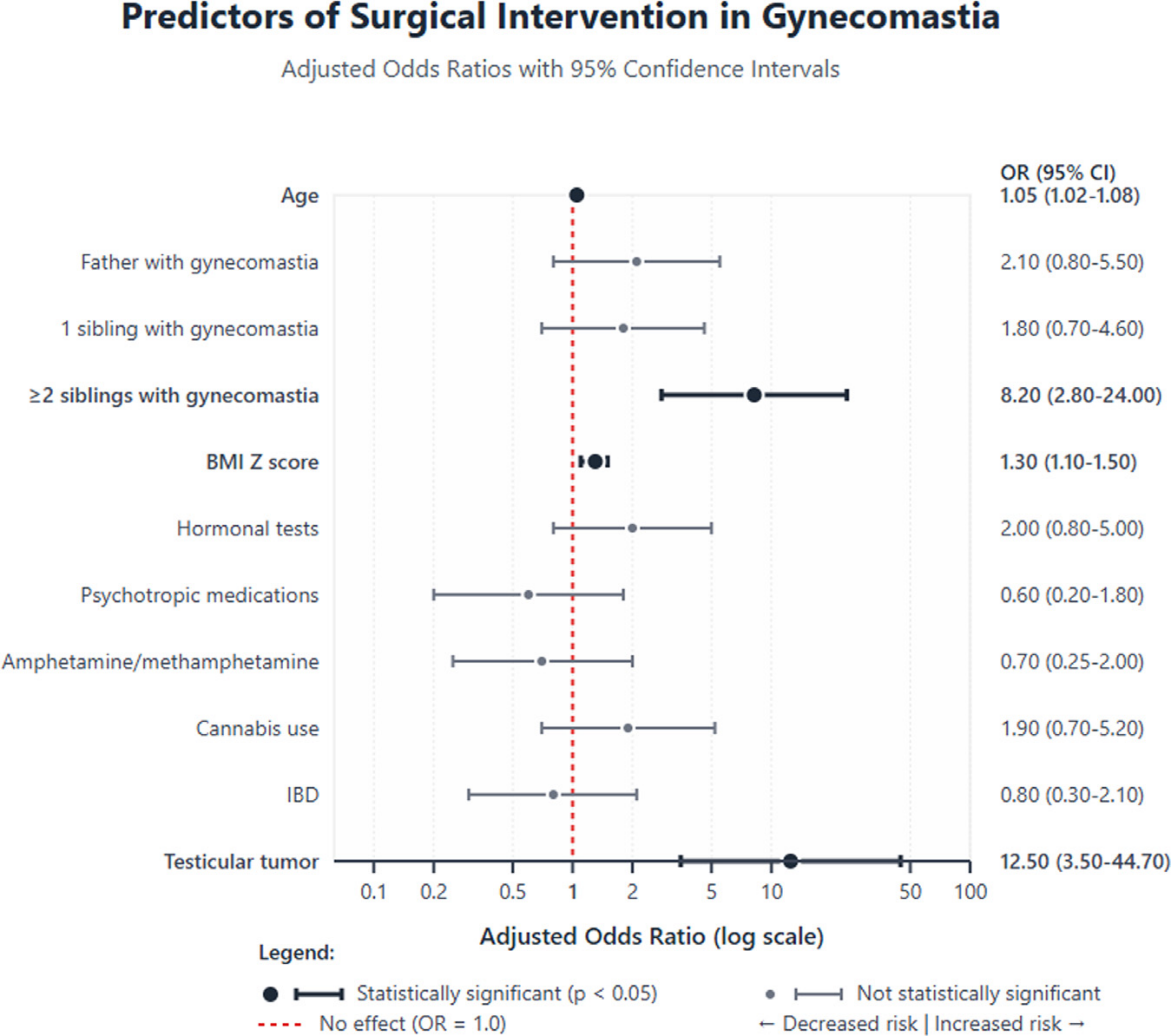
Associations of patient characteristics with surgical interventions for gynecomastia.

Surgery was performed in 676 patients (3.4%), at a mean 0.82 ± 6.15 months after diagnosis. For patients who underwent surgery, the mean age was older (16.78 ± 3.64 vs. 15.56 ± 3.55; *p* < 0.001) and the mean BMI Z-score was higher (1.51 ± 1.17 vs. 0.89 ± 1.45; *p* < 0.001). Family history was more common (father: *p* < 0.001; sibling: *p* < 0.001; > 2 siblings: *p* = 0.006). Hypogonadism (4% vs. 1.4%; *p* < 0.001) and hyperthyroidism (1.3% vs. 0.5%; *p* = 0.016) were more frequent. Cannabis use was higher (2.1% vs. 0.5%; *p* < 0.001). Variables associated with surgery included older age (adjusted OR 1.204; 95% CI 1.143–1.268; *p* < 0.001), higher BMI Z-score (adjusted OR 1.300; 95% CI 1.033–1.635; *p* = 0.025), and ≥2 siblings affected (adjusted OR 8.352; 95% CI 1.970–35.408; *p* = 0.004). Testicular tumor was found to be associated with surgery (adjusted OR 14.055; 95% CI 3.082–64.104; *p* < 0.001). The model discrimination was excellent (area under the curve =0.973, Figure 2).

### Obesity and gynecomastia

**Figure 3:**
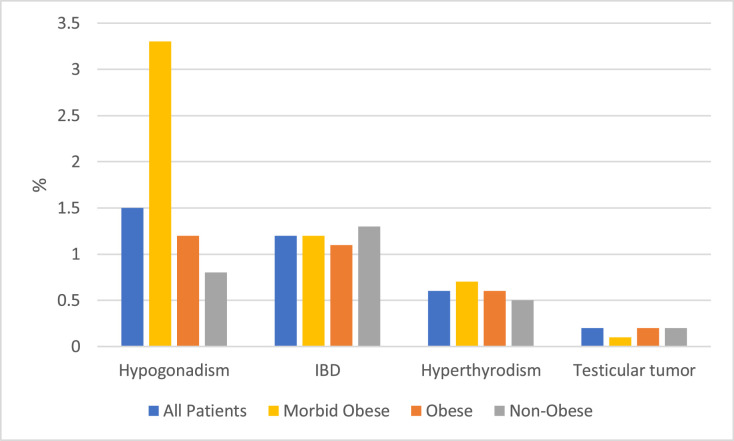
Prevalences of comorbidities according to body weight categories: morbid obesity, obesity and without obesity.

Among the cohort, 4929 (25.1%) were with morbid obesity, 4691 (23.9%) with obesity, and 10,020 (51%) without obesity. The mean ages were 14.9, 15.8, and 15.8 years, respectively (*p* < 0.001). The respective mean frequencies of sibling gynecomastia were 5.3%, 4.2%, and 3.5%; *p* < 0.001; and of hypogonadism, 3.3%, 1.2%, and 0.8%. Psychiatric medication, cannabis, amphetamine, and methamphetamine use were more common among those with morbid obesity. Surgical intervention was most frequent in those with morbid obesity (5.2%) versus obesity (4.4%) and without obesity (2.1%, *p* < 0.001; Table 5, Figure 3).

**Table 5:** Demographic, clinical, and etiologic characteristics of patients with gynecomastia, stratified by body weight categories: morbid obesity, obesity and without obesity.

Characteristics		Morbid obesity (*N* = 4929)	Obesity (*N* = 4691)	Without obesity (*N* = 10,020)	*p* value
Age at diagnosis (years), Mean ± SD		14.93 ± 3.39	15.82 ± 3.70	15.83 ± 3.53	<0.001
BMI. Mean ± SD		34.05 ± 4.20	24.47 ± 2.45	19.57 ± 2.69	<0.001
BMI Z-score Mean ± SD		2.73 ± 0.60	1.45 ± 0.28	− 0.23 ± 0.92	<0.001
Family history of gynecomastia					
	Father with gynecomastia	101 (2)	80 (1.7)	162 (1.6)	0.160
	1 sibling with gynecomastia	263 (5.3)	197 (4.2)	348 (3.5)	<0.001
	>2 siblings with gynecomastia	18 (0.4)	21 (0.4)	25 (0.2)	0.124
Alcohol use		12 (0.2)	9 (0.2)	32 (0.3)	0.350
Drug use		9 (0.2)	6 (0.1)	16 (0.2)	0.794
Medications					
	Hormonal and endocrine	38 (0.8)	36 (0.8)	99 (1)	0.260
	Psychotropic	261 (5.3)	204 (4.3)	367 (3.7)	<0.001
	Cardiovascular	12 (0.2)	18 (0.4)	24 (0.2)	0.264
	Chemotherapeutic	0 (0)	1 ()	4 ()	0.348
	Amphetamine and methamphetamine	1096 (22.2)	900 (19.2)	1917 (19.1)	<0.001
	Cannabis	33 (0.7)	31 (0.7)	48 (0.5)	0.223
Hormonal tests		4681 (95)	4398 (93.8)	9033 (90.1)	<0.001
Surgery		258 (5.2)	207 (4.4)	211 (2.1)	<0.001
Months from diagnosis to surgery		1.33 ± 7.95	1.11 ± 7.20	0.43 ± 4.3	<0.001

**Figure 4:**
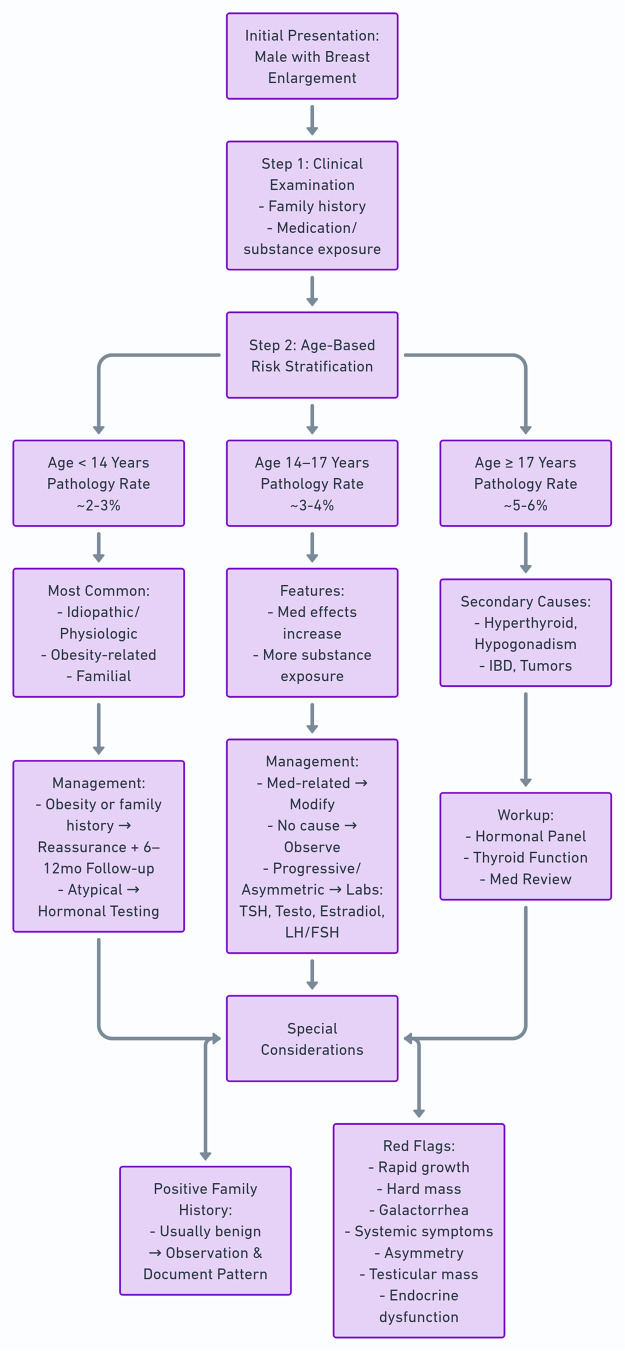
A gynecomastia management algorithm based on our results.

Figure 4 presents a gynecomastia management algorithm based on our results.

## Discussion

This study, one of the largest pediatric and young adult gynecomastia cohorts reported, demonstrates clear age-dependent variation in etiology and clinical presentation. These findings emphasize the need for risk-based, age-sensitive diagnostic and management strategies rather than a uniform approach. Gynecomastia patterns differed substantially across adolescence, reflecting the developmental, endocrine, and exposure-related transitions that have been described.^10^

Nearly two-thirds of younger children (10–11 years) met criteria for obesity, compared with less than half of young adults aged 20–25 years. This compares with reports that adolescents with obesity tended to present earlier with gynecomastia, likely due to increased aromatase activity and leptin-mediated endocrine effects.^4^ Our findings align with previous evidence that familial gynecomastia is more commonly identified during early adolescence, when pubertal hormonal changes amplify inherited susceptibilities.^5^^,^^11^ These patterns suggest that physiologic, obesity-related, and familial etiologies dominate early adolescence, while secondary causes become more common later in development. In our cohort, this transition became apparent from approximately 17 years of age, when the prevalence of pathology-associated gynecomastia increased compared with younger age groups.

Medication and substance exposures, including psychotropic agents, cannabis, and stimulants, increased in frequency with age. These trends parallel population-level adolescent behavior patterns^12^^,^^13^ and are consistent with known endocrine and dopaminergic mechanisms linking psychotropic medications and cannabis to gynecomastia.^7^^,^^14^ Clinically, this underscores the importance of detailed drug and substance histories, particularly in mid-to-late adolescence, when non-physiologic contributors become increasingly relevant. However, these associations should be interpreted cautiously, as the study design does not distinguish causal from coincidental medication exposure. Observed drug use may reflect confounding by indication, whereby patients with more complex medical conditions are more likely to receive multiple medications, as well as surveillance bias due to closer monitoring of medically complex patients. Similarly, although alcohol use reached statistical significance, its clinical relevance in this cohort remains uncertain.

Pathology-associated gynecomastia occurred more often in older adolescents and young adults, consistent with existing literature showing that systemic and endocrine disorders typically manifest later in puberty.^15^ Hypogonadism, hyperthyroidism, and IBD were more common in patients aged ≥14 years, indicating a lower threshold for endocrine evaluation in this age group. Conversely, testicular tumor-related gynecomastia was more prevalent in younger adolescents, consistent with the known hormonal activity of Leydig and Sertoli cell tumors.^16^^,^^17^ This age-dependent dichotomy supports stratified diagnostic pathways that emphasize metabolic and familial factors in early adolescence, and that expand to endocrine, systemic, pharmacologic, and substance-related etiologies in older adolescents.

Surgical intervention was relatively uncommon but was more likely among older adolescents and young adults and was independently associated with older age, higher BMI Z-score, multiple affected siblings, and testicular tumors. These associations should be interpreted in their clinical context, as surgical decisions in gynecomastia are often patient-driven and influenced by cosmetic and psychosocial considerations rather than medical necessity. However, it is important to note that most patients receive authorization for gynecomastia surgery only after age 16 years. This may introduce bias when interpreting the association between age and surgical intervention. Only select patients are approved for surgery before age 16 years, based on medical necessity and physician discretion. Furthermore, some patients may have undergone surgical correction in private clinics without follow-up documentation in the database, thus suggesting that the true number of surgically treated patients is likely underestimated. Although the model’s high area under the curve (0.973) raises the theoretical possibility of overfitting, the very large cohort and adequate number of surgical events mitigate this concern. Nevertheless, external validation would strengthen generalizability.

Obesity was strongly associated with earlier onsets of gynecomastia, higher comorbidity rates, and increased likelihood of surgical evaluation. The hormonal effects of adiposity, including increased aromatization and estrogen-testosterone imbalance, likely contribute to both true glandular gynecomastia and pseudo-gynecomastia.^18^

Genetic and environmental factors, including those related to aromatase activity and body composition, may also contribute, particularly among patients with morbid obesity.^19^^,^^20^ These findings emphasize the importance of early endocrine assessment, lifestyle counseling, and medication review in adolescents with obesity.

Overall, this study supports age-specific diagnostic algorithms and highlights the need for focused evaluation of obesity, family history, medication exposure, and endocrine disease when assessing gynecomastia in adolescents and young adults.

### Strengths and limitations

This study has several limitations. Its retrospective, registry-based design limits causal inference and may introduce misclassification, particularly in distinguishing true gynecomastia from pseudo-gynecomastia in patients with obesity. These findings should be interpreted cautiously, as reliance on clinical examination alone may have led to overestimation of glandular gynecomastia, particularly in patients with obesity. Important confounders, including Tanner stage and socioeconomic factors were unavailable, and this may contribute to residual confounding. Operative details and postoperative outcomes were inconsistently recorded. However, as surgical management was not the primary aim of this study, these omissions do not affect the core objective of identifying the adolescents who may benefit from endocrine evaluation before considering surgery.

Although the dataset derives from a single healthcare system, Clalit Health Services serves a large and heterogeneous population including Jewish, Arab, Russian-speaking people, and other minority groups. This supports strong internal validity. Nevertheless, patients managed within tertiary centers may differ from those treated in the community, regarding referral patterns, healthcare access, and health-seeking behavior, all of those included in our study. Socioeconomic and ethnic differences, and culturally influenced attitudes toward cosmetic versus endocrine evaluation, may affect presentation and management. Therefore, while the findings are likely generalizable within the Israeli healthcare context, differences in healthcare systems and cultural norms between Israel and other countries may limit external applicability.

## Conclusion

This large-scale, population-based study highlights the dynamic, age-dependent etiologies of pediatric and young adult gynecomastia. Idiopathic and obesity-related etiologies dominated in younger males, while medication- and pathology-associated gynecomastia were more common in older adolescents and young adults. A positive family history generally suggested a benign course, though caution is warranted in early puberty (ages 12–13 years). Obesity significantly influenced presentation, surgical intervention rates, and endocrine comorbidities, thus emphasizing the importance of early metabolic counseling. Age-sensitive, risk-based diagnostic assessment screening for obesity and family history in early adolescents and expanding evaluation to pharmacologic, substance-related and systemic causes in older patients can refine diagnostic pathways. For plastic surgeons, incorporating these tailored strategies can better identify patients who may benefit from endocrine evaluation before considering surgery. This may reduce unnecessary testing, optimize operative planning, and improve outcomes for children and adolescents presenting with gynecomastia.

## Funding

None.

## Data availability declaration

The data that support the findings of this study are available from the corresponding author upon reasonable request.

## Declaration of competing interest

The author has no conflicts of interest to declare.

## Appendix Supplementary materials

Image, application 1
